# Retrograde Migration of a Percutaneous Endoscopic Gastro-Jejunal Tube Into the Esophagus

**DOI:** 10.7759/cureus.52105

**Published:** 2024-01-11

**Authors:** Binyamin R Abramowitz, Jude Noel, Sushil Ahlawat

**Affiliations:** 1 Gastroenterology, State University of New York Downstate Health Sciences University, New York, USA; 2 Gastroenterology and Hepatology, State University of New York Downstate Health Sciences University, New York, USA

**Keywords:** aspiration pneumonia, tube feeds, tube migration, enteral nutrition, gastrojejunostomy, percutaneous endoscopic gastro-jejunostomy, percutaneous endoscopic gastrostomy

## Abstract

Percutaneous endoscopic gastrostomy (PEG) and percutaneous endoscopic gastro-jejunal (PEG-J) tube placement are both common procedures regularly performed on patients requiring nutritional support. These procedures may be complicated by infection, hemorrhage, fistulization, or tube migration. We present an extremely rare case of a patient with a PEG-J tube that migrated into the esophagus.

## Introduction

Percutaneous endoscopic gastrostomy (PEG) and percutaneous endoscopic gastro-jejunal (PEG-J) tube placement are commonly performed on patients requiring long-term enteral nutritional support. Due to underlying pathology, these patients have a mismatch between their oral caloric intake and their overall metabolic demands [1]. Pathological disease processes causing this mismatch include stroke, dementia, motor neuron disease, gastrointestinal malignancies, and prolonged coma [2,3]. However, the progressively worsening clinical courses of these pathologies are not the sole determining factor as to whether PEG or PEG-J tube placement is indicated. Rather, detailed discussions about the goals of care are required. These discussions aim to elucidate the prognosis, priorities, and realistic expectations of the patient before finalizing the decision on whether tube feeding is indicated [4].

While PEG tubes are inserted through the abdominal wall to deliver feeds directly into the stomach, PEG-J tubes are inserted similarly but with an extension leading into the jejunum so that feeds bypass the stomach itself and are directed into the small bowel. Although PEG tubes are more common, patients may still be at high risk for aspirating gastric feeds, especially in conditions such as gastric outlet obstruction, gastroparesis, and gastric resection [5,6]. In many of these patients, PEG-J tubes may be a better option, as feeds are delivered more distally into the jejunum, thereby reducing the risk of aspiration [7]. The placement of a PEG-J tube is uncommonly complicated by tube migration, which can lead to infection, hemorrhage, fistulization, buried bumper syndrome, or aspiration pneumonia [8,9]. We present a unique case of a PEG-J tube that was found to have migrated into a patient’s mid-esophagus.

## Case presentation

A 29-year-old female with a past medical history of sickle cell disease, cerebral palsy, and cerebral vascular accident status post-PEG-J tube placement presented with her PEG-J tube clogged since the previous day. The PEG-J tube had last been replaced only four months earlier. Although the patient was non-verbal at baseline, she appeared comfortable and not in any acute distress. Her mother reported no vomiting, fever, or chills, and no aberrant behavior from the patient’s baseline. She was hemodynamically stable: blood pressure of 112/78, heart rate of 92 beats per minute, respiratory rate of 18 breaths per minute, temperature of 98.0˚F, and oxygen saturation at 98% on room air. Of note, she had recently been discharged from a three-month-long hospitalization for sepsis secondary to pneumonia complicated by pulmonary embolism treated with both antibiotics and full-dose anticoagulation.

On physical examination, the patient’s lungs were clear to auscultation bilaterally with no wheezes or crackles, and her abdomen was soft and non-tender. The PEG-J tube site appeared clean, non-erythematous, and with the tube properly in place. Laboratory data showed a white blood cell count of 9.74 K/uL, hemoglobin of 9.9 g/dL, platelets of 186 K/uL, and all electrolytes as well as renal function within normal limits. The chest X-ray was largely unremarkable, though it was noted to be a limited study due to the non-ideal positioning of the patient. Although the PEG-J tube site appeared clean, the balloon lumen appeared to be hyperinflated, and both the jejunal as well as the gastric ports could not be flushed properly, as every flush attempt resulted in saline pouring right back out of the tube. Attempts to unclog the tube with ginger ale were unsuccessful.

**Figure 1:**
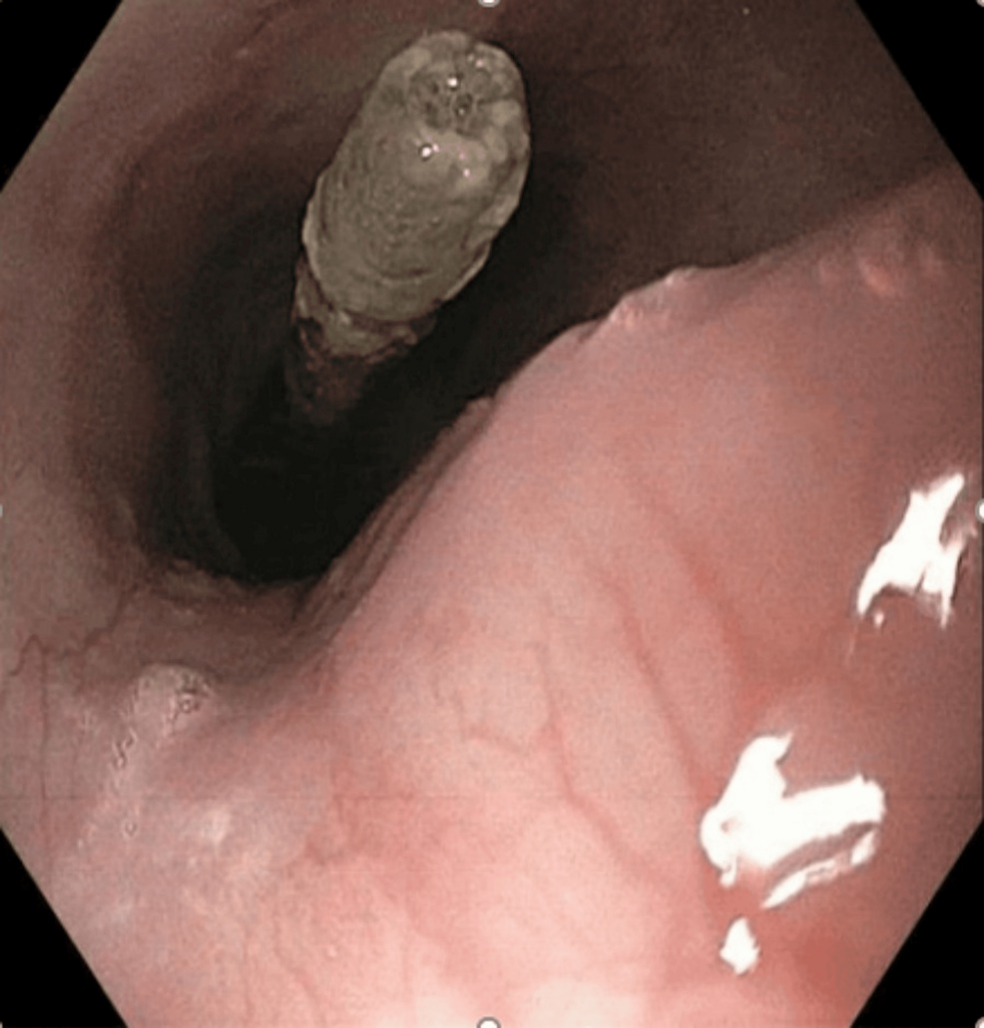
Jejunal limb of PEG-J tube within the mid-esophagus PEG-J: Percutaneous endoscopic gastro-jejunal

**Figure 2:**
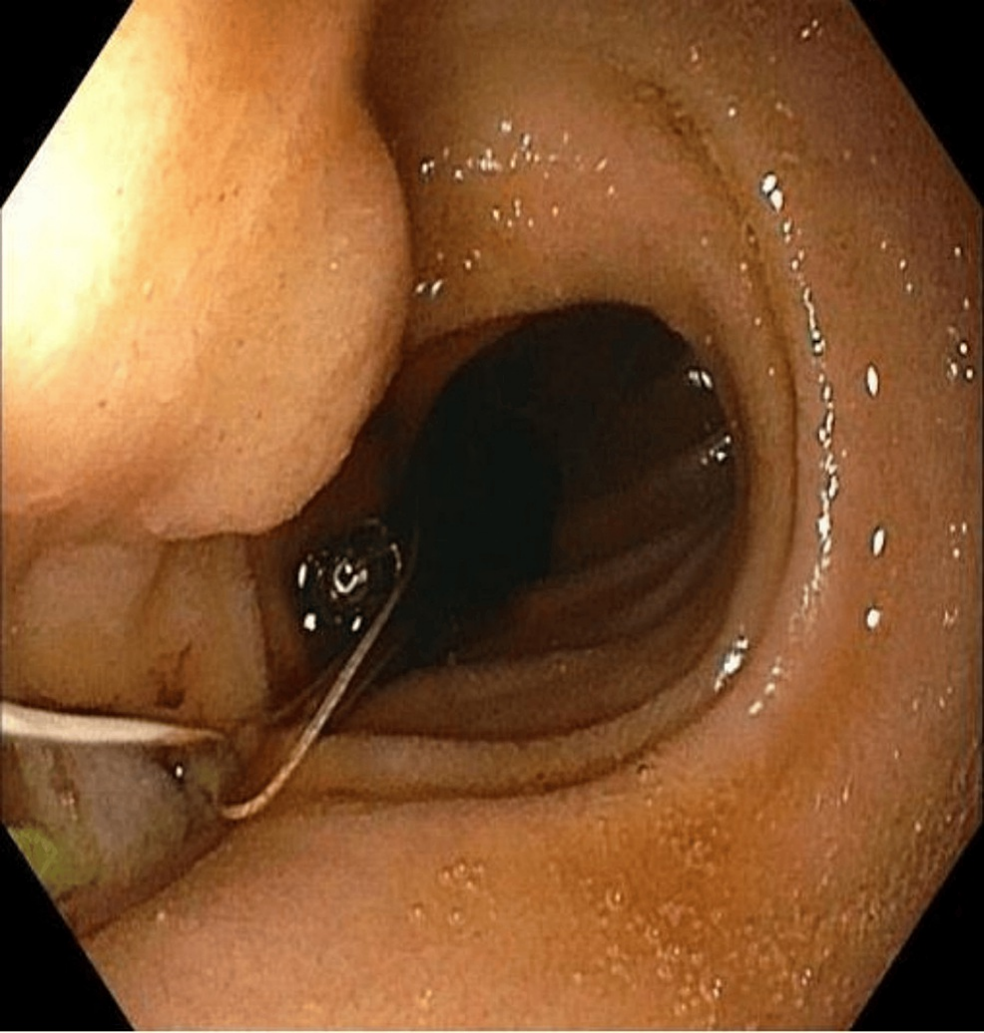
Jejunal limb of new PEG-J tube anchored with endoscopic clips PEG-J: Percutaneous endoscopic gastro-jejunal

The gastroenterology service was consulted, and after further unsuccessful attempts to unclog the tube, esophagogastroduodenoscopy (EGD) was subsequently performed for tube replacement. During the procedure, the distal tip of the PEG-J tube was visualized to be within the mid-esophagus (Figure 1). Following balloon deflation, the tube was removed. Using the prior tube tract, a new 24-French gastrostomy (G) tube was placed, utilizing the endoscope as well as a guidewire. A jejunostomy (J) tube was then placed via the G tube under endoscopic guidance. The J tube was subsequently anchored in the second portion of the duodenum using endoscopic clips and surgical sutures (Figure 2). After feeds were initially restarted, the patient was unable to tolerate continuous feeding as she was having episodes of minimal emesis once the feeding rate exceeded 40 cc/hr. The patient was switched to bolus feeds, which she tolerated well with no episodes of emesis or leakage from the tube. After several days of tolerating bolus feeding, the patient was safely discharged.

## Discussion

Tube migration is a known complication of PEG-J tube placement and is associated with negative outcomes such as aspiration, sepsis, hemorrhage, and fistulization. A retrospective study of PEG-J tubes placed using endoscopic clips and surgical sutures found that 7% of all tube placement was complicated by migration [10]. Anterograde migration rarely occurs when sutures anchoring the tube in place erode through the skin, leading to tube dislodgement [11]. Physiologic peristalsis subsequently pushes the tube more distally into the small bowel and, in some cases, can even get into the colon [12]. In contrast, the retrograde migration of PEG-J tubes into the stomach itself is well documented in the literature [13]. A recent retrospective review found that tube insertion into the gastric body rather than the antrum and tube entry tracts directed away from the pylorus rather than toward it were both associated with a greater likelihood of retrograde migration of the jejunal limb into the stomach [14].

Retrograde migration of the jejunal limb of PEG-J tubes into the esophagus is extremely rare in the adult population, with only two reported cases in the literature [15,16]. In both cases, proximal tube migration was associated with vomiting and aspiration. As feeds are exiting the tube more proximally into the esophagus instead of the small bowel, the feeds being in closer proximity to the respiratory tract puts the patient at a much higher risk of aspiration. Although our patient only presented with a malfunctioning PEG-J tube rather than the acute vomiting or aspiration pneumonia presentation that has previously been reported, it is definitely worth noting that the patient had recently been discharged from a three-month-long hospitalization for sepsis secondary to pneumonia. In retrospect, it is likely that retrograde migration of the PEG-J tube occurred months before the patient’s current presentation and may have been a triggering event for aspiration that resulted in hospitalization for pneumonia and sepsis.

## Conclusions

This case report describes the retrograde migration of a PEG-J tube into the esophagus, highlighting an extremely rare complication of PEG-J tubes. This complication can have various presentations; it may involve persistent gagging or vomiting, the development of aspiration pneumonia, or it can simply present as an inability for the tube to flush properly. While the distal placement of PEG-J tubes is designed to prevent feed aspiration, it does not completely eliminate the risk. Retrograde migration is one potential complication that significantly increases the risk of aspiration. Tube migration into the esophagus should be within the differential diagnosis for patients with PEG-J tubes presenting with aspiration pneumonia.
